# Coagulation abnormalities in childhood acute lymphoblastic leukemia: assessing the impact of L-asparaginase therapy in Ghana

**DOI:** 10.1186/s12959-021-00297-4

**Published:** 2021-06-26

**Authors:** William Osei-OWusu, David Ofosu Ntiamoah, Gordon Asare Akuffo, Selina Mintaah, Michael Owusu, Benedict Sackey, Lilian Antwi-Boateng, Ganiwu Abdul, Max Annani-Akollor, Eddie-Williams Owiredu, Alexander Yaw Debrah, Otchere Addai-Mensah

**Affiliations:** 1 Department of Medical Diagnostics, Faculty of Allied Health Sciences, Kwame Nkrumah University of Science and Technology, Kumasi, Ghana; 2 Department of Basic and Applied Biology, University of Energy and Natural Resources, Sunyani, Ghana; 3 Department of Molecular Medicine, School of Medicine and Dentistry, Kwame Nkrumah University of Science and Technology, Kumasi, Ghana

**Keywords:** Acute lymphoblastic leukemia, Hypercoagulability, Thrombosis, Thromboembolic complications

## Abstract

**Background:**

Although the rate of childhood acute lymphoblastic leukemia (ALL) is increasing in Africa, there is a dearth of information on the disease and the dynamics of hemostatic parameters with therapy.

**Methods:**

In this case-control study, we evaluated variations in the level/activity of selected coagulation parameters among cALL in Ghana and healthy controls stratified by stage of therapeutic management.

**Results:**

In all, the research recruited 104 participants comprising 26 cALL cases and 78 healthy controls. The cALL group had significantly higher prothrombin time (PT) (*p* = 0.001), activated partial thromboplastin time (APTT) (*p* < 0.0001) and D-dimers (*p* = 0.001) but lower platelet (PLT) count, protein C (PC) (*p* < 0.0001), protein S (PS) (*p* < 0.0001) and antithrombin III (ATIII) (*p* < 0.0001) compared to controls. Compared to the healthy controls, activity of PC was lower during induction (*p* < 0.0001), consolidation (*p* = 0.005) and maintenance phases of chemotherapy (*p* = 0.012) while activities of PS and ATIII were lower at both induction (*p* < 0.0001, *p* = 0.006) and consolidation (*p* < 0.0001, *p* = 0.018) phases of chemotherapy.

**Conclusion:**

Our findings provide evidence in the context of Africa and corroborates previous reports that cALL could result in a state of hypercoagulability, possibly leading to a high risk of thrombosis and thromboembolic complications. This possibly increased risk is not limited to the induction phase but also the consolidation phase.

**Supplementary Information:**

The online version contains supplementary material available at 10.1186/s12959-021-00297-4.

## Introduction

Acute lymphoblastic leukemia (ALL) is a common leukemia that occurs predominantly in children. Evidence suggest that over 60% of ALL occurs at pediatric age [1]. Childhood acute lymphoblastic leukemia (cALL) is associated with life-threatening thromboembolic complications which may complicate the course of chemotherapy with a negative prognostic impact. The incidence of thrombosis is reported to vary between 1 and 36% [1, 2]. The wide range of the incidence rate is attributed to the different definitions of thrombosis, diagnostic methods used, and differences in chemotherapy protocols [3]. The risks of the thromboembolic-associated complications have been reported to be linked with the disease itself or therapeutic management, where the risk is higher during the induction phase of chemotherapy [3].

L-asparaginase is the principal chemotherapeutic agent used in cALL and it is often administered in combination with a corticosteroid, vincristine and intrathecal chemotherapy [4–6]. Since its standardization as a component of ALL treatments, L-asparaginase has dramatically improved the outcome of ALL particularly in adults; however, the overall survival in cALL remains low [5]. Mechanistically, following induction of chemotherapy, L-asparaginase hydrolysis asparagine to L-aspartic acid and ammonia, resulting in reduced blood levels of free asparagine. The asparagine depletion primarily affects lymphoblasts due to their limited capacity to produce asparagine as a result of low asparagine synthetase activity, hence the effectiveness of L-asparaginase in rapidly impairing lymphoblast protein secretion, cellular function and increasing cell death [7].

This notwithstanding, L-asparaginase also affects proteins essential for hemostatic regulation. L-asparaginase reduces the synthesis of fibrinogen, protein C (PC), protein S (PS), antithrombin III (ATIII) [8]. This side effect of L-asparaginase could augment the risk of thrombotic events in cALL by contributing to hypercoagulable state as reported by previous studies [8–12]. Furthermore, coagulation parameters vary between therapeutic protocol due to different combinations of drugs of different types or origin and differences in the chronology of drug administration [2]. Therefore, the risk of aberrant hemostatic regulation (which is affected by geographic and genetic variations) and the associated risk of thrombosis can vary; nonetheless, reports in the African context, despite increasing rate of the diseases in children (ALL is reported to comprise about 50–80% of childhood leukemia cases across the African continent despite lack of adequate diagnostic which leads to under-diagnosis [13]), is limited. In this case-control study among children from Ghana, we evaluated the levels of D-dimers and the activity of ATIII, PC, and PS in cALL compared with healthy controls and further assessed the impact of the different stages of chemotherapeutic management**.**

## Methods

### Ethical clearance

Ethical approval for this study was obtained from the institutional review board of the Komfo Anokye Teaching Hospital (KATH) (RD/CR19/187) and the management of the Pediatric Oncology Unit, KATH. Participation was voluntary and written informed consent was obtained from parents/guardians of each participant prior to enrollment.

### Study design/site and population

This case-control study was conducted at the Pediatric Oncology Unit of KATH in Kumasi. KATH is a center of excellence and referral teaching hospital established to serve the Ashanti, Western, Western North, Ahafo, Bono East, Bono, Eastern, Central and the 5 Northern regions of Ghana and to train students from the College of Health Sciences at the Kwame Nkrumah University of Science and Technology (KNUST). One hundred and four (104) children (comprising 26 ALL cases and 78 apparently healthy controls) who were < 15 years old were purposively recruited for the study. Children with medical history of renal and/or hepatic impairment, sickle cell disease or malaria were excluded from the study. In our inclusion criteria for the selection of control subjects, participants with no malaria, sickle cell disease and a normal ESR were recruited as control participants.

### Blood sample collection

Six milliliters (6 ml) of venous blood was obtained from each participant under aseptic conditions. Three to four milliliters of this blood was dispensed into EDTA tubes for complete blood count, blood film commenting, sickle cell test, hemoglobin electrophoresis and malaria parasites screening for all participants. Erythrocyte sedimentation rate was done for control subjects to rule out possible underlying inflammatory conditions. The remainder 2-3mls of blood was dispensed into tubes containing sodium citrate (3.8% W/V) for the estimation of prothrombin time (PT), activated partial thromboplastin time (APTT) and activities of ATIII, PC and PS.

### Laboratory analysis

Complete blood count was assessed using XN 2000 fully automated Sysmex Hematology (Sysmex Corporation, Kobe, Japan) according to the manufacturer’s instructions. Sickle cell test and hemoglobin electrophoresis were performed using the 2% sodium metabisulphite technique and electrophoresis at alkaline pH, respectively. Malaria parasite screening was performed by microscopy. Ten percent on Giemsa-stained thick and thin films were prepared on clean grease-free slides. Erythrocyte sedimentation rate was done using the Westergren method.

Platelet poor plasma, obtained by spinning whole blood in the tube containing sodium citrate for 15 min at 304.4×g at room temperature, was used for the estimation of PT and APTT using the CoaREAD 2a semi-automated coagulation analyzer (AXIOM CoaRead 2a 2-channel coagulometer, Germany). Activity of ATIII, PC and PS was evaluated using the solid phase sandwich ELISA (Melson Co. Ltd., China) according to the manufacturer’s protocol.

Briefly, standards, samples and controls were pipetted into pre-coated microtitre, mixed with the enzyme conjugate reagent, sealed and incubated at 37 °C for 60 min. Then, chromogen was added and incubated at 37 °C for 15 min. The reaction was stopped with Stop Solution. The absorbance of the final colored product was measured spectrophotometrically at 450 nm using Thermo Electron Multiskan EX plate reader (Shanghai, China), the activity of analyte estimated using standard curve generated from the mean OD450 for each reference standard.

### Statistical analysis

Categorical data were presented as frequencies (percentages). For continuous data, normality was checked using Shapiro-Wilk’s test and visual inspection with Q-Q plots. Normally distributed data were presented as mean ± SD and significance of differences were assessed between the cases and controls using independent t-tests. One-way analysis of variance (ANOVA) with Tukey post hoc multiple comparisons tests were used to assess significance of differences of markers between controls and various treatment groups. Nonparametric data were presented as median (interquartile ranges) and significance of differences were evaluated using Mann-Whitney U tests and Kruskal-Wallis W tests with Dunn post hoc multiple comparison tests, where applicable. Multiple comparisons were adjusted using Bonferroni correction. Statistical analysis was performed using Prism 8 version 8.02. All tests were two-sided and *p*-value < 0.05 was considered statistically significant.

## Results

A total of 78 children with mean age of 8.62 ± 3.61 years old and 26 children with mean age of 9.88 ± 2.86 years old were included as controls and cases, respectively. Six (23.1%) and 7 (26.9%) of the cases presented with lymphadenopathy and hepatomegaly, respectively. Among the cases, 4 (15.4%) were newly-diagnosed whereas 7 (26.9), 4 (15.4) and 11 (42.3) were in the induction, consolidation and maintenance phases of chemotherapy (Table 1). Additionally, the cases had significantly lower red blood cell count (*p* < 0.0001), hemoglobin levels (*p* < 0.0001), hematocrit (*p* < 0.0001), total white blood cell count (*p* = 0.001), absolute neutrophil count (*p* = 0.003), absolute lymphocyte count (*p* < 0.0001), absolute monocyte count (*p* = 0.003), absolute eosinophil count (*p* = 0.043), absolute basophil count (*p* < 0.0001) and basophil % (*p* = 0.042) but higher mean cell volume (*p* = 0.012) and mean cell hemoglobin (*p* = 0.019) compared to the controls (Additional file 1).

**Table 1:** Baseline characteristics of the study population

Variables	Control (*n* = 78)	Case (*n* = 26)	*p*-value
Age (yrs)/mean ± SD	8.62 ± 3.61	9.88 ± 2.86	0.107
Sex			1.000*
Females/n (%)	38 (48.7)	12 (46.2)	
Males/n (%)	40 (51.3)	14 (53.8)	
Weight (Kg) /mean ± SD	29.28 ± 11.21	30.19 ± 8.75	0.671
Height (m) /mean ± SD	1.27 ± 0.22	1.35 ± 0.15	0.025
BMI(Kg/m^2^) /mean ± SD	17.52 ± 1.84	16.16 ± 2.15	0.002
Lymphadenopathy	0 (0.0)	6 (23.1)	< 0.0001*
Hepatomegaly	0 (0.0)	7 (26.9)	< 0.0001*
Treatment			NA
Newly diagnosed	–	4 (15.4)	
On treatment	–	22 (84.6)	
*Induction*	*–*	*7 (26.9)*	NA
*Consolidation*	*–*	*4 (15.4)*	NA
*Maintenance*	*–*	*11 (42.3)*	NA

Unless otherwise indicated, statistical analyses were performed using independent t-tests. *: statistical analysis was done using Chi-squared/Fisher’s exact test. *NA* Non-applicable

**Fig. 1:**
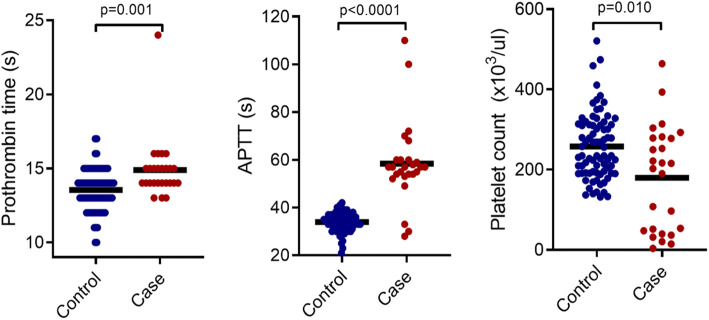
Comparison of PT, APTT and platelet count between controls and cases. Comparison of PT (**A**), APTT (**B**) and platelet count (**C**) between cases and controls are displayed as scatter plots. Significance of differences were determined using Mann-Whitney U tests. *P* < 0.05 was considered statistically significant

The cases presented with significantly higher PT (15.04 ± 2.79 s vs 13.54 ± 1.40s, *p* = 0.001) and APTT (57.00 (53.75–60.00)s vs 34.00 (31.75–36.25)s, *p* < 0.0001) but lower platelet count (209.50 (46.00–279.75) × 10^3^/μl vs 235.00 (191.75–312.00) × 10^3^/μl, *p* = 0.01) compared to the controls (Fig. 1).

**Fig. 2:**
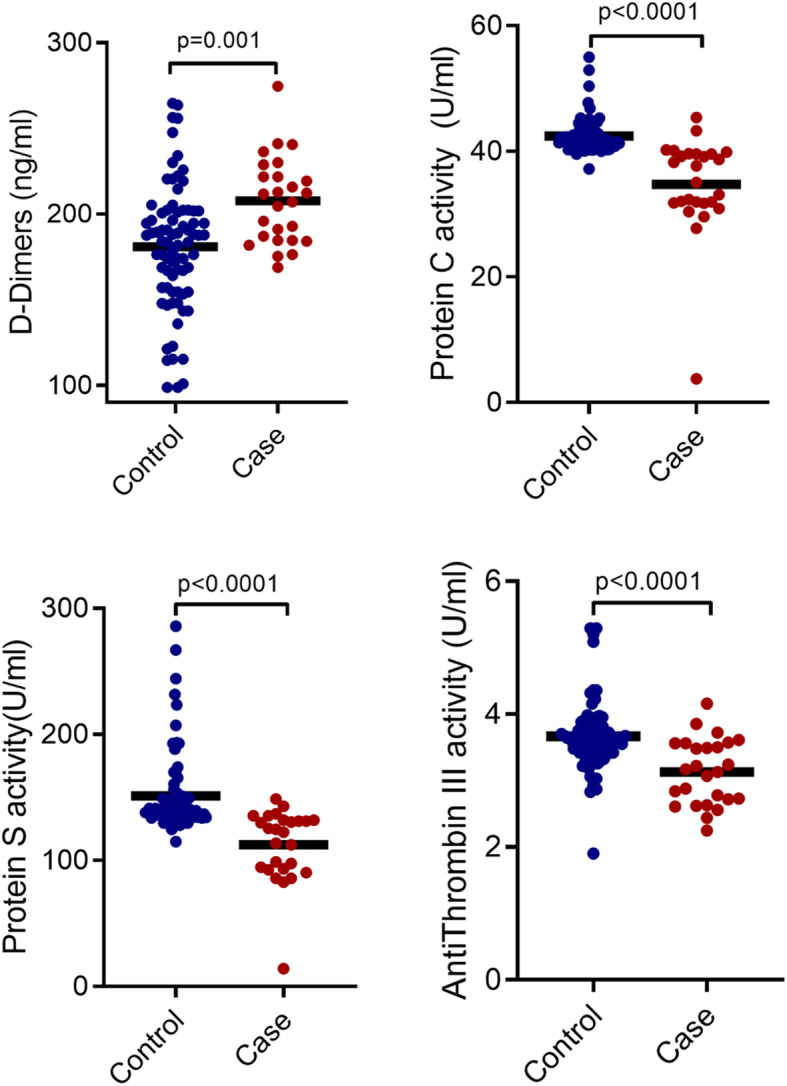
Comparison of D-dimer levels, PC, PS and AT-III activity between controls and cases. Comparison of D-dimer (**A**), PC activity (**B**) PS activity (**C**) and (**D**) ATIII activity between cases and controls are displayed as scatter plots. Significance of differences were determined using Mann-Whitney U tests. *P* < 0.05 was considered statistically significant

Significantly higher levels of D-dimer (*p* = 0.001) but lower activity of PC (*p* < 0.0001), PS (*p* < 0.0001) and ATIII (*p* < 0.0001) were observed among the cases compared to the controls (Fig. 2).

### Comparison of PT, APTT and platelet count between controls, newly-diagnosed and treatment groups

Participants on treatment had significantly higher PT (*p* = 0.037) and APTT (*p* < 0.0001) compared to the control group, and higher PLT count (*p* = 0.037) compared to the newly-diagnosed group. The newly-diagnosed group had significantly higher APTT (*p* = 0.002) but lower PLT count (*p* = 0.002) compared to the control group (Fig. S1; Additional file 1).

### Comparison of D-dimer levels, protein C, protein S and antithrombin III activity between controls, newly-diagnosed and treatment groups

Participants on treatment presented with significantly lower D-dimer levels (*p* = 0.026) and PC activity (*p* < 0.0001) compared to the newly-diagnosed group, and lower PS activity (*p* < 0.0001), PC activity (*p* < 0.0001) and ATIII activity (*p* < 0.0001) compared to the control group. On the other hand, the newly-diagnosed group had significantly higher D-dimer levels (*p* = 0.009) but lower PC activity (*p* = 0.01) compared to the controls (Fig. S2; Additional file 1).

**Fig. 3:**
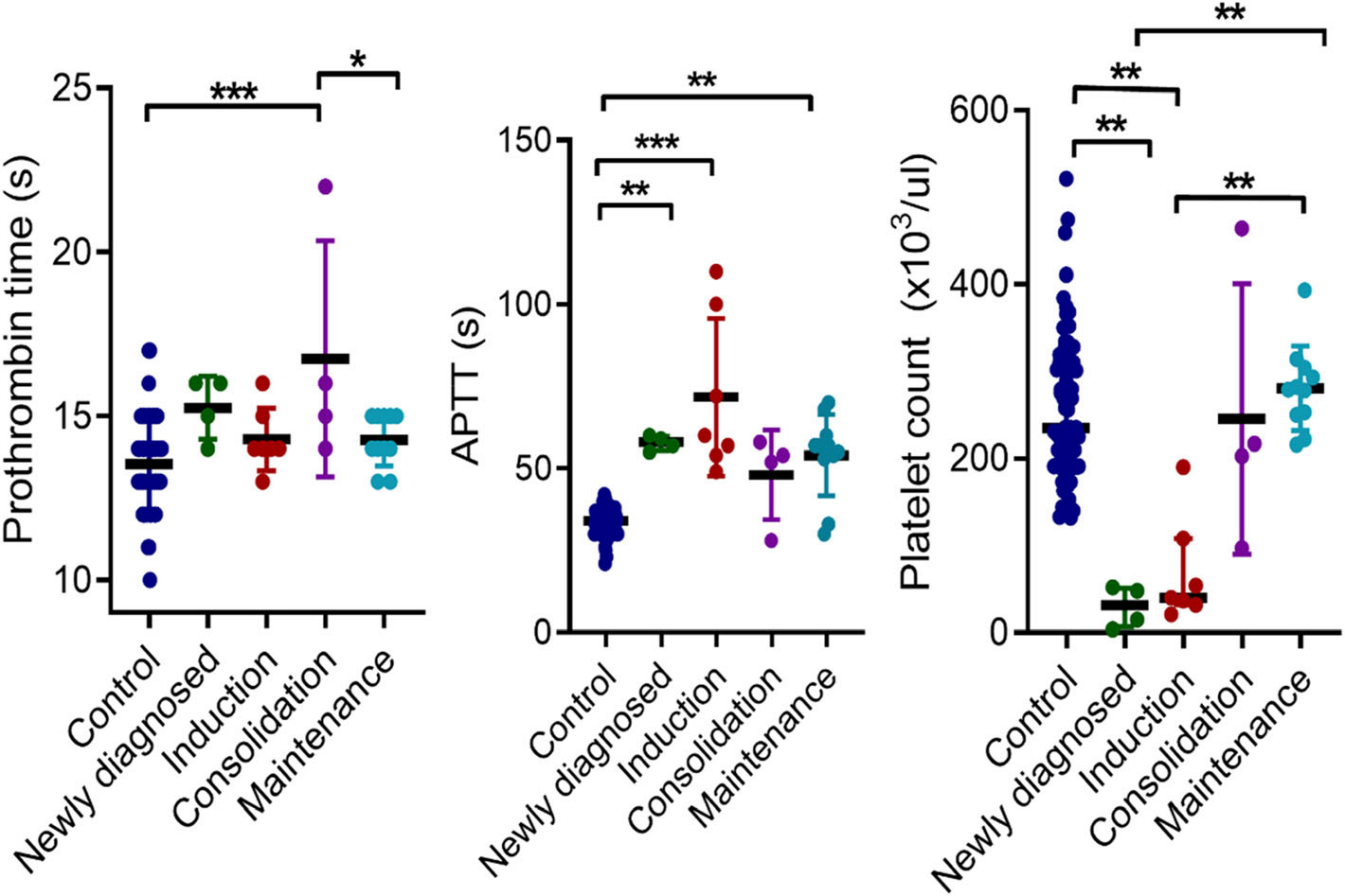
Comparison of prothrombin time, activated partial thromboplastin time and platelet count between controls, newly-diagnosed and treatment-stratified groups. Comparison of PT (**A**), APTT (**B**) and platelet count (**C**) between the three groups are displayed as scatter plots. Significance of differences were determined using Kruskal-Wallis W with Dunn post hoc multiple comparison tests. *P* < 0.05 was considered statistically significant. *; *p* < 0.05, **; *p* < 0.01, ***; *p* < 0.0001

Participants in the consolidation phase of chemotherapy had significantly higher PT compared to those in the maintenance phase (*p* = 0.031) and the controls (*p* < 0.0001). Participants in the induction (*p* < 0.0001) and maintenance (*p* = 0.001) phases of chemotherapy had significantly higher APTT compared to the controls, respectively. Contrarily, patients in the induction phase had significantly lower PLT count (*p* = 0.001) compared to the controls. Participants in the maintenance phase had significantly higher PLT counts compared to the newly-diagnosed (*p* = 0.003) and the induction phase (*p* = 0.001), respectively (Fig. 3).

**Fig. 4:**
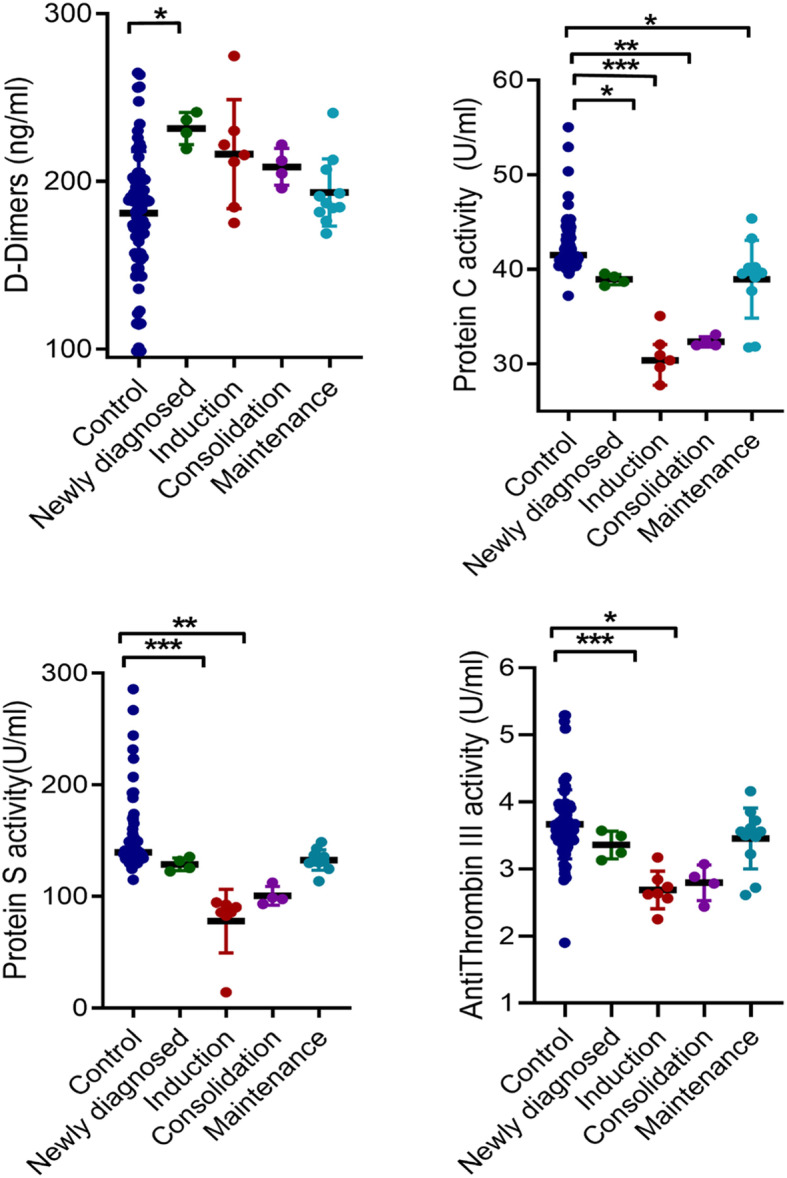
Comparison of D-dimers, protein C, protein S and antithrombin III between controls, newly-diagnosed and treatment-stratified group. Comparison of D-dimer (**A**), PC activity (**B**) PS activity (**C**) and (**D**) ATIII activity between cases and controls are displayed as scatter plots. Significance of differences were determined using Kruskal-Wallis W with Dunn post hoc multiple comparison tests. *P* < 0.05 was considered statistically significant. *; *p* < 0.05, **; *p* < 0.01, ***; *p* < 0.0001

The levels of D-dimer was not significantly affected upon stratification by treatment groups. However, PC activity was significantly lower in patients in the induction (*p* < 0.0001), consolidation (*p* = 0.005) and maintenance phases of chemotherapy (*p* = 0.012) compared to the controls, respectively. Similarly, PS and ATIII activities were significantly lower in patients in both the induction and consolidation phases compared to the control group (Fig. 4).

## Discussion

This study reports a deranged coagulation profile among Ghanaian children with ALL (cALL). Patients with ALL had higher PT and APTT but lower platelet count when compared to apparently healthy controls. The higher PT and APTT in cALL could be attributed to chemotherapy-associated reduction coagulation factors (mainly fibrinogen) synthesis by the liver as previously suggested by Bushman et al. [7]. During primary hemostasis, platelets adhere to exposed vWF to facilitate clot formation and contribute to the cessation of bleeding [14]; thus, the reduced platelet count, which may possibly be attributed to the effect of blast infiltration into the bone marrow, implies that cALL could predispose to higher bleeding tendencies. The prolongation of PT and APTT reflects derangements in both intrinsic and extrinsic coagulation pathways which altogether, further corroborates the fact that cALL are more prone to bleeding tendencies. Since PT and APTT characterize blood coagulation based on the extrinsic and intrinsic pathways, respectively, our findings suggest a relationship between cALL and coagulopathy with both pathways likely being affected.

ALL has however been previously shown to be associated with increased risk of thrombosis [2, 8, 10, 12] which seem to contradict the increased bleeding risk suggested that the above findings would suggest. We therefore investigated whether the risk of thrombosis could be as a result of limited activity of the natural anticoagulants such as PC, PS and ATIII, which play significant roles in limiting superfluous generation of thrombin after the activation of the coagulation pathways. Contrary to the evidence of increased bleeding tendencies, we found that the activities of ATIII, PC and PS were lower in cALL compared to controls. This finding corroborates with the previous reports and suggests that cALL may be associated with increased risk of thrombosis by limiting the activity of the natural anticoagulants [2, 8, 10, 12]. Furthermore, D-dimer, whose level is suggestive of previous coagulation incidences or an on-going activation of the hemostatic system, was higher among children with ALL. The higher D-dimer levels could be a result of the impact of tissue factor-rich microparticles and cancer procoagulants produced by leukaemic cells on the coagulation system as reported by Gheldof et al. [15]. Similar findings have been reported by previous studies. A study by Sehgal et al. [12] reported lower activity of PS and PC compared to controls at baseline. Dixit et al. reported reduced levels of PC, PS and ATIII in 43, 57 and 17% of ALL patients in India [16]. Jalali et al. [17] found lower activity of ATIII and PC in Iranian patients with ALL compared to healthy controls. Together with previous reports, our findings suggest that children with ALL may be at a higher risk of thrombotic events relative to the healthy population.

There is evidence that risk of thrombosis in ALL is either linked with the disease itself or therapeutic management [8]. To test this in our study cohort, we first stratified the cases group into newly diagnosed (yet to initiate treatment) and those who were already on treatment. We found that although newly-diagnosed cALL group had higher D-dimers compared to the treatment group, those on treatment had lower PC, PS and ATIII activity compared to the controls. Furthermore, the activities of PS and ATIII were comparable between the newly diagnosed and the controls. This finding implicates chemotherapy as a factor for hemostatic abnormality in cALL.

Our treatment group were heterogenous, comprising 26.9, 15.4 and 42.3% children in the induction, consolidation and maintenance phases, respectively. Thus, to investigate which stage of chemotherapy is associated with the hypercoagulabity observed among the treatment group, we restratified the treatment group into their corresponding subgroups. When compared to healthy controls, the activity of PC was lower at all stages of chemotherapy; however, the induction phase had with the most deranged hemostatic parameters, presenting with higher APTT but lower activity of ATIII, PS and PC. The reduced activity of the natural anticoagulants during the induction phase was expected as it could be due to greater side effects of L-asparaginase which is administered in high dose during induction. Although the primary role of L-asparaginase is the hydrolysis and subsequent reduction in asparagine levels, and leveraging on the low asparagine synthetase activity in lymphoblasts to impair protein secretion, cellular function and increasing cell death following induction of chemotherapy, L-asparaginase also affects proteins essential for hemostatic regulation [7, 10]. This side effect of L-asparaginase could account for the reduced activity of the natural anticoagulants. Furthermore, given the high D-dimer levels, the reduced activities of PS, PC and ATIII could also be attributed to the consumption of the anticoagulants due to subclinical coagulation activation and also partly due the hepatotoxicity associated with high doses chemotherapy resulting in decrease protein synthesis by the liver [12]. It is however worth noting that, contrary to previous reports that the risk of thrombosis in ALL occur in the induction phase of treatment, although the activity of the natural anticoagulants (ATIII, PC, PS) was lowest in the induction phase, it was low to a comparable extent in the consolidation phase as well. Thus, the risk of thrombosis in the consolidation phase should not be underestimated. It is however also possible that the low activity of the anticoagulants during the consolidation phase is due to residual effect of L-asparaginase administered during the induction, and that our findings were affected by the low number of patients in consolidation phase in our sample.

## Conclusions

In conclusion, childhood ALL is associated with an increased in PT, APTT plasma D-dimer levels but lower PLT count, activities of PC, PS and ATIII. The derangement was higher during the induction phase of chemotherapy. Our findings provide evidence in the context of Africa and corroborates previous reports that cALL could result in a state of hypercoagulability, possibly leading to a high risk of thrombosis and thromboembolic complications. This possibly increased risk is not limited to the induction phase but also the consolidation.

## Supplementary Information

**Additional file 1 Table S1.** Comparison of hematological profiles of controls and cases.

## Data Availability

The datasets supporting the conclusions of this article are included within the article and its additional file.

## Abbreviations

cALL: Childhood acute lymphoblastic leukemia
PC: Protein C
PS: Protein S
ATIII: Antithrombin III
