# Supplementary material for: Coagulation abnormalities in childhood acute lymphoblastic leukemia: assessing the impact of L-asparaginase therapy in Ghana
Source: Thromb J. 2021 Jun 26;19:44. doi: 10.1186/s12959-021-00297-4 (PMC8235643; doi:10.1186/s12959-021-00297-4)
Supplement: Supplementary file 1 — Additional file 1 Table S1. Comparison of hematological profiles of controls and cases. [file 12959_2021_297_MOESM1_ESM.docx]

**Coagulopathy in childhood acute lymphoblastic leukemia: assessing the impact of therapeutic management**

William Osei-OWusu^1^, David Ofosu Ntiamoah^2^, Gordon Asare Akuffo^1^, Selina Mintaah^1^, Michael Owusu^1^, Benedict Sackey^1^, Lilian Antwi-Boateng^1^, Ganiwu Abdul^1^, Max Annani-Akollor^3^, Eddie-Williams Owiredu^3^, Alexander Yaw Debrah^1^, Otchere Addai-Mensah^1,*^

**Table S1. Comparison of haematological profiles of controls and cases**.

| **Variables** | **Case** | **Control** | **p-value** |
| --- | --- | --- | --- |
| RBC (10^6^/µl) | 3.22±0.76 | 4.54±0.41 | <0.0001 |
| Haemoglobin (g/dl) | 9.02±1.98 | 12.15±1.07 | <0.0001 |
| HCT (%) | 25.71±5.78 | 34.47±2.57 | <0.0001 |
| MCV (fl) | 80.19±5.00 | 76.35±7.08 | 0.012 |
| MCH (pg) | 28.25±2.10 | 26.91±2.60 | 0.019 |
| MCHC (g/dl) | 35.23±1.39 | 35.89±5.63 | 0.556 |
| TWBC (10^3^/µl) | 3.87 (1.89-7.12) | 5.50 (4.62-7.83) | 0.001 |
| Neutrophil (%) | 41.75 (18.50-53.45) | 39.80 (32.30-48.75) | 0.964 |
| Neutrophil Abs (10^3^/µl) | 1.46 (0.51-2.75) | 2.27(1.66-3.55) | 0.003 |
| Lymphocyte (%) | 40.85 (32.95-58.05) | 45.10 (38.50-54.20) | 0.566 |
| Lymphocyte Abs (10^3^/µl) | 1.32 (0.76-1.99) | 2.45 (2.03-3.25) | <0.0001 |
| Monocyte (%) | 9.10 (4.98-15.40) | 9.30 (7.57-11.10) | 0.682 |
| Monocyte Abs (10^3^/µl) | 0.33 (0.13-0.65) | 0.54 (0.45-0.76) | 0.003 |
| Eosinophil (%) | 1.80 (0.00-3.55) | 2.10 (1.00-3.13) | 0.57 |
| Eosinophil Abs (10^3^/µl) | 0.08 (0.01-0.15) | 0.12 (0.04-0.23) | 0.043 |
| Basophil (%) | 0.25 (0.00-0.50) | 0.40 (0.20-0.50) | 0.042 |
| Basophil Abs (10^3^/µl) | 0.01 (0.00-0.02) | 0.02 (0.01-0.04) | <0.0001 |

Independent t-tests and Mann-Whitney U tests were used to compare case and controls for normally distributed data (showed as mean±SD) and non-parametric data (showed as median (interquartile ranges)), respectively. P-values <0.05 was considered statistically significant. RBC: Red Blood Cells; HCT: Haematocrit; MCV: Mean Cell Volume, MCH: Mean Cell Haemoglobin; MCHC: Mean Cell Haemoglobin Concentration; TWBC: Total White Blood Cells; Abs: Absolute.


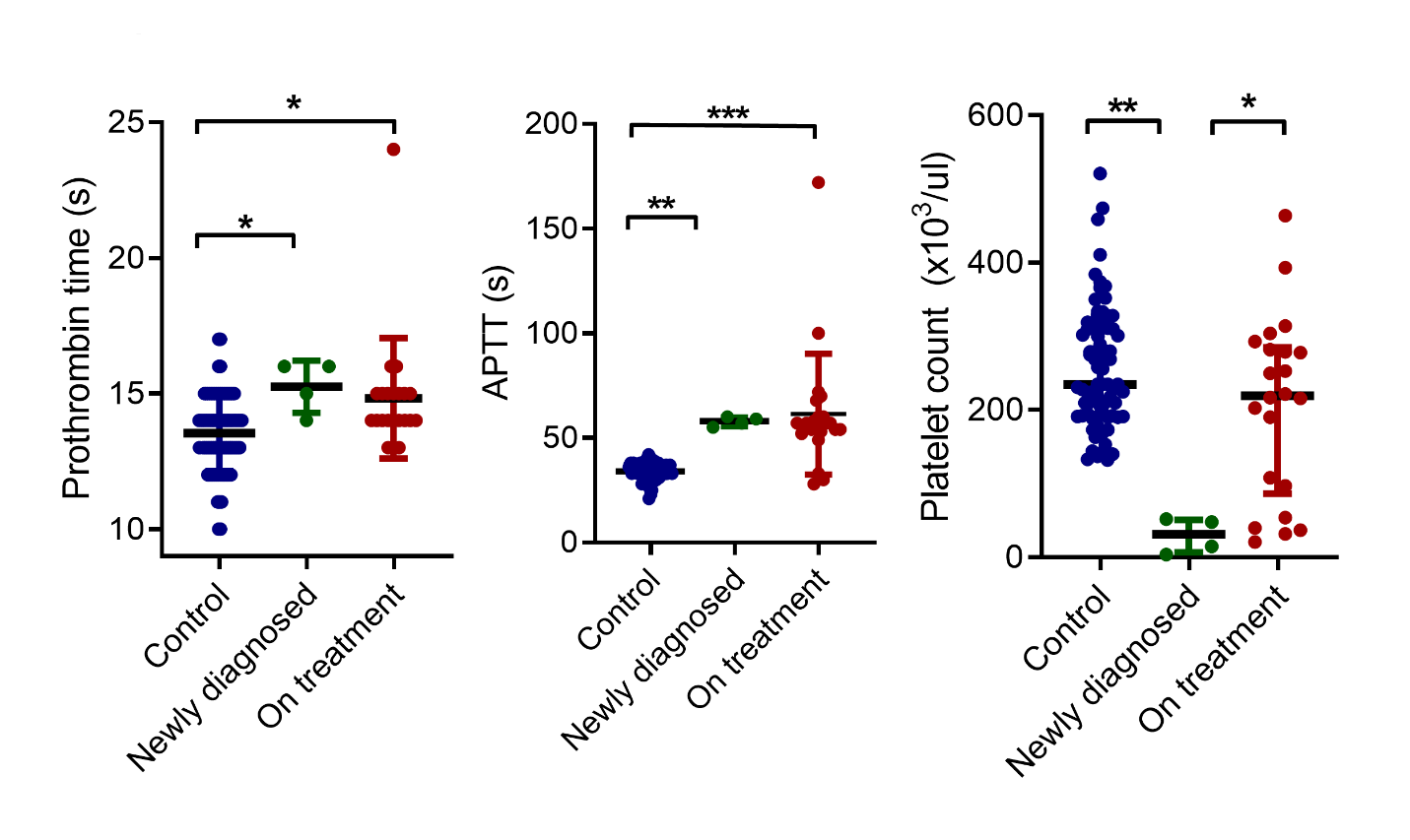


**Figure S1. Comparison of PT, APTT and platelet count between controls, newly-diagnosed and treatment groups.** Comparison of PT (A), APTT (B) and platelet count (C) between the three groups are displayed as scatter plots. Significance of differences were determined using Kruskal-Wallis W with Dunn post hoc multiple comparison tests. P <0.05 was considered statistically significant. *; p<0.05, **; p<0.01, ***; p<0.0001


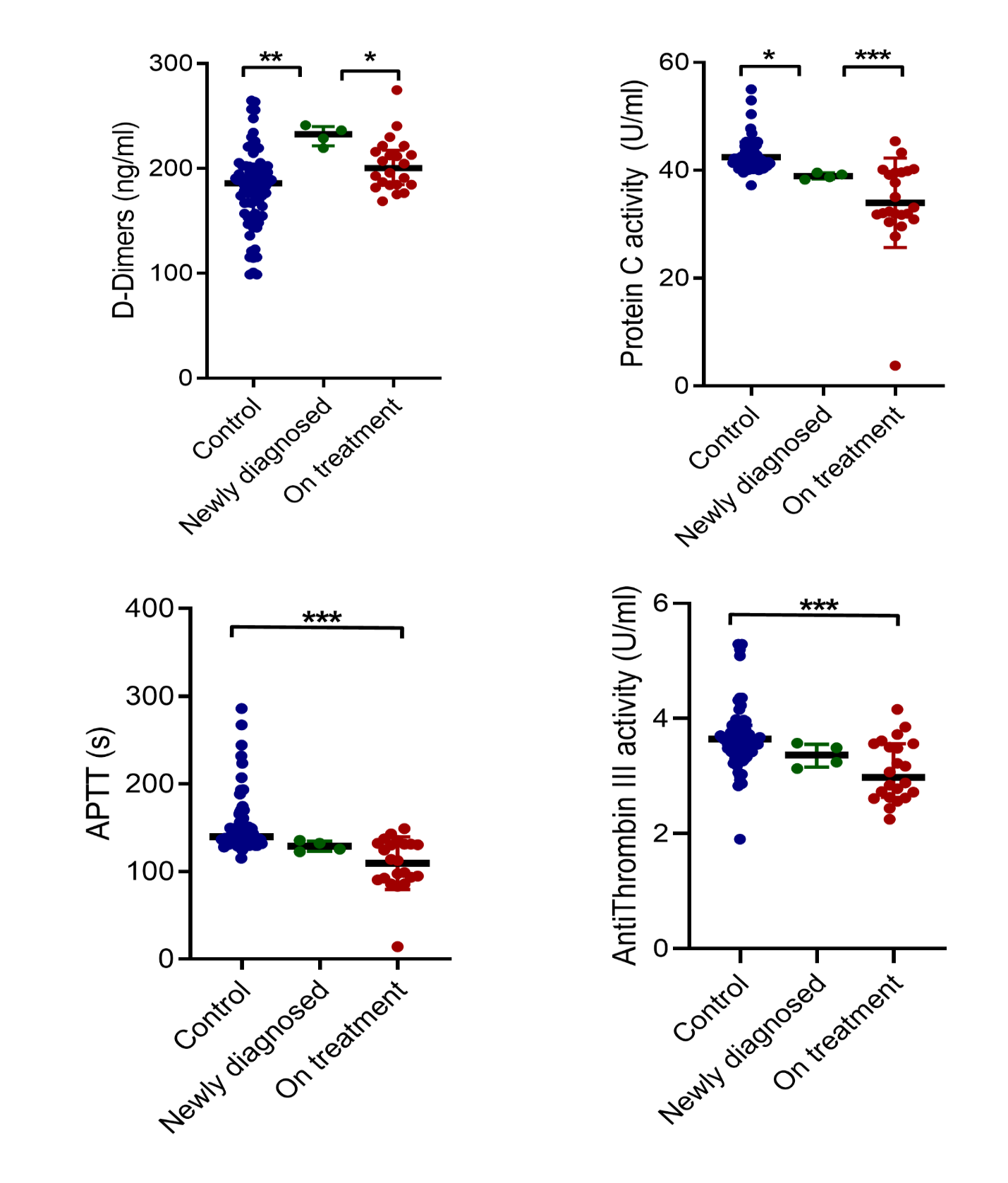


**Figure S2. Comparison of D-dimer levels, protein C, protein S and antithrombin III activity between controls, newly-diagnosed and treatment groups.** Comparison of D-dimer (A), PC activity (B) PS activity (C) and (D) ATIII activity between cases and controls are displayed as scatter plots. Significance of differences were determined using Kruskal-Wallis W with Dunn post hoc multiple comparison tests. P <0.05 was considered statistically significant. *; p<0.05, **; p<0.01, ***; p<0.0001
